# The Potential Benefits of HPV Vaccination in Previously Infected Women

**DOI:** 10.1016/j.ebiom.2016.08.005

**Published:** 2016-08-03

**Authors:** John T. Schiller

**Affiliations:** Center for Cancer Research, National Cancer Institute, NIH, United States

HPV vaccines are highly effective at preventing anogenital HPV infections and the neoplastic diseases that they cause (Herrero et al., 2015). Like other licensed anti-viral prophylactic vaccines, the HPV vaccines are thought to function primarily by inducing antibodies that bind the virus, thereby preventing infection (Schiller and Lowy, 2012). In the case of the three licensed HPV vaccines, these antibodies are induced by antigens comprised of L1 virus-like particles (VLPs), which morphologically and immunologically resemble the outer shell of the authentic virus. Cervarix, Gardasil, and Gardasil-9 are based on VLPs of two, four, and nine HPV types, respectively. The ability of the VLPs to consistently induce robust and durable systemic virus neutralizing antibody responses after intramuscular injection almost certainly explains their high type-specific efficacy against infection in clinical trials and effectiveness in national vaccination programs. In contrast to vaccination, natural HPV infections inconsistently induce virion antibody responses, usually after a delay of several months. The responses are typically several orders of magnitude lower than after vaccination, and are detected only transiently in some individually (Carter et al., 2000). The effectiveness of the antibodies induced by natural infection in preventing reinfection by the same HPV type remains controversial, although there is some evidence for protection in women, at least those with relative high titers of circulating antibodies (Beachler et al., 2016).

Given this background, the study of Scherer et al. reported in this issue (Scherer et al., 2016) is of considerable interest, because it provides the first in-depth analysis of the change in the VLP/virion antibody repertoire in individual women who were naturally infected by HPV, and who are then vaccinated with VLPs. The authors used state-of-the-art technologies to isolate circulating virion-specific memory B cells (Bmem). They then sequence the antibody-encoding genes, and finally clone, express, and functionally test the antibodies they encode. The primary, and perhaps surprising, conclusion of the study is that the antibodies in the Bmem repertoire after natural infection are predominantly low avidity and non-neutralizing. In contrast, the Bmem repertoire in women who were boosted by a single dose of Gardasil mostly contains higher avidity and neutralizing antibodies. The authors previously reported that women who were seronegative at the time of vaccination also generated mostly neutralizing antibodies (Scherer et al., 2014). The findings support the possibility that women who are seropositive due to infection might nonetheless benefit from VLP vaccination. However, it is important to note that the authors appropriately caution that they conducted “an exploratory, unblinded pilot study”, and public health policy should not be unduly influenced by the tentative conclusions drawn from a study of five individuals. Further studies are warranted.

This study highlights several important questions in this field. The first question is why the antibody response to infection and vaccination appear to be so qualitatively different. Given the small number of virion-binding Bmem identified after infection, it is difficult to eliminate the possibility of confounding due to the inclusion of Bmem that were actually specific for another antigen but sufficiently cross-reactive toward VLPs to be isolated using the procedures employed. Arguing against this scenario is the fact that the same isolation procedure identified mostly high avidity and neutralizing clones after vaccination. A far more attractive possibility is that the difference reflects the very different context in which the antigen is presented to the immune system after infection versus vaccination. With intramuscular vaccination, the antigen is delivered at high dose to the systemic immune system in a pro-inflammatory context, due to the presence of the adjuvant. This type of exposure of a repetitive particulate antigen generally induces a strong germinal center reaction in the draining lymph nodes that in turn generates hypersomatic mutations in the variable domains of the immunoglobulin genes and ultimately high avidity antibodies (Eisen, 2014). In contrast, productive HPV infections are limited to epithelial surfaces and are largely non-inflammatory. In addition, L1 protein is expressed and viruses are produced only in the most superficial epithelial layers. Therefore, during infection, L1 will usually be exposed to the immune system at very low dose and predominately in a non-inflammatory setting, a situation that would seem unlikely to induce a long lived germinal center reaction (Stanley and Sterling, 2014). Antibodies generated in this context would be expected to have the low avidity characteristics observed in the virion antibodies detected after infection. However, this otherwise attractive explanation is difficult to reconcile with the authors' finding that the number of somatic mutations in the L1 antibodies sequenced was similar before and after vaccination. Clearly there are aspects of the B cell responses to virus-like antigens, particularly in a low dose mucosal context, that require further investigation.

A second question is whether the circulating Bmem examined in the study play an active role in protection from HPV infection or neoplastic disease. Neutralizing serum antibodies produced by long lived plasma cells (LLPCs), which reside mainly in the bone marrow, are almost certainly the primary mediators of protection. Bmem are the reserve forces of serologic memory that produce antibodies only after reexposure to antigen and so could produce antibodies only after infection had occurred. It is uncertain whether a new infection in a mucosal epithelium would generate enough virion antigen to activate systemic Bmen in a timely fashion and if it did, whether it would prevent neoplastic progression, for instance by preventing subsequent rounds of auto-innoculation or new transmissions from an infected partner.

If Bmem play, at best, a minor role in protection, then a third important question is whether the VLP-specific antibody repertoire in Bmem largely predicts the one in LLPCs. It was recently shown that most Bmem are generated in an early period after germinal center formation, while most LLPCs are generated at a later time (Weisel et al., 2016). This timing predicts that the antibodies produced by LLPCs will tend to have more somatic mutations. Emerging data suggest that the two repertoires may be quite distinct. For instance, a recent study coupling high-resolution mass spectrometry-based proteomic analysis of circulating antibodies and high throughput sequences of immunoglobulin genes in Bmem after tetanus toxoid booster vaccination of human subjects concluded the IgG clones in Bmem were more diverse and largely distinct from those expressed by LLPCs (Lavinder et al., 2014). As a follow-up to Sherer et al., it would be of great interest to use a similar approach to compare the L1 antibody repertoires in LLPCs and Bmem after and VLP vaccination, and if technically feasible, after natural infection.

## Conflict of Interest Statement

JTS is an inventor of U.S. government-owned patents that have been licensed to Merck and GlaxoSmithKline, the manufacturers of the commercial HPV vaccines.
